# 217 closed Salmonella reference genomes using PacBio sequencing

**DOI:** 10.1186/s12863-025-01304-7

**Published:** 2025-02-28

**Authors:** Yan Luo, Jae Hee Jang, Maria Balkey, Maria Hoffmann

**Affiliations:** 1 U.S. Food and Drug Administration, Human Foods Program, College Park, MD USA; 2 U.S. Department of Agriculture, Agriculture Research Service, Beltsville, MD USA

**Keywords:** Pacbio sequencing, Closed genomes, Diverse Salmonella serovars

## Abstract

**Objectives:**

Whole Genome Sequencing (WGS) is widely used in food safety for the detection, investigation, and control of foodborne bacterial pathogens. However, the WGS data in most public databases, such as the National Center for Biotechnology Information (NCBI), primarily consist of Illumina short reads which lack some important information for repetitive regions, structural variations, and mobile genetic elements, and the genomic location of certain important genes like antimicrobial resistance genes (AMR) and virulence genes. To address this limitation, we have contributed 217 closed circular *Salmonella enterica* genomes that were generated using PacBio sequencing to the NCBI Pathogen Detection (PD) database and GenBank. This dataset provides a higher level of accuracy to genome representations in the database.

**Data description:**

High-quality complete reference genomes generated from PacBio long reads can provide essential details that are not available in draft genomes from short reads. A complete reference genome allows for more accurate data analysis and researchers to establish connections between genome variations and known genes, regulatory elements, and other genomic features. The addition of 217 complete genomes from 78 different Salmonella serovars, each representing either a distinct SNP cluster within the NCBI PD database or a unique strain, significantly enriches the diversity of the reference genome database.

## Objective

In 2012, the U.S. Food and Drug Administration’s Center for Food Safety and Applied Nutrition (FDA-CFSAN) launched the GenomeTrakr network [1], the first distributed network of laboratories that utilize WGS for foodborne pathogen identification. GenomeTrakr data is submitted to the NCBI PD database to assist in foodborne pathogen surveillance and outbreak detection. To date, WGS data of over 1.7 million isolates belonging to 81 different pathogen species, primarily obtained from Illumina short-read sequencers, has been collected, stored, and processed in NCBI. The NCBI PD web portal (https://www.ncbi.nlm.nih.gov/pathogens/) provides public access to this extensive collection of WGS data. This portal offers high resolution strain typing, outbreak investigation, and surveillance capabilities. However, the NCBI PD database currently contains only around 500 closed genomes indicating a shortage of high-quality complete genomes.

Salmonella, one of the leading foodborne pathogens, causes widespread illnesses and poses a significant threat to public health worldwide. To address the need for more comprehensive genomic information, between 2018 and 2021 we focused on sequencing and completely closing a diverse set of *Salmonella* genomes using PacBio technology. This effort provides higher resolution, accuracy, and precision to various analyses, including the detection of structural variations, gene annotations, phylogenetic analysis, and comparative genomics. These analyses which are often used during foodborne events require critical information that is often lacking in the short-read assemblies. The 217 Salmonella isolates used in this collection were collected from various food sources, clinical, and environment. Both the closed genomes and raw long reads have been deposited in NCBI (Table 1), contributing to the expansion of the available genomic resources for *Salmonella* surveillance.

## Data description

NCBI PD processes the WGS data from bacterial and fungal pathogen genomes and places them into clusters based on the relatedness between genomes. Specifically for Salmonella, approximately 711,000 genomes were assigned to more than 30,000 clusters as of January 20, 2025. Within each cluster, a reference genome was used to construct a reference-based SNP matrix, which in turn was used to infer a phylogenetic tree. Having a high-quality closed reference genome would greatly help to identify lineage or serovar-specific Salmonella fragments, enhance the accuracy of the phylogenetic analysis, SNP annotation, and other related analyses [2, 3]. This enhanced resolution helps trace the source of the outbreaks more accurately, determine the pathways of contamination, and understand the genetic factors involved in virulence and resistance [4–6]. Consequently, it will lead to more effective and targeted interventions to control and prevent future outbreaks, ensuring better food safety and public health protection [7]. For our study we carefully selected 217 Salmonella isolates for PacBio sequencing. Each isolate represents either a distinct NCBI SNP cluster or is a unique isolate that did not cluster with any other genomes.

Each isolate was cultured in Luria-Bertani (LB) medium at 35 ºC overnight. DNA was extracted using the Maxwell RSC Cultured Cell DNA kit. The sequencing libraries were generated using the SMRTbell Template Prep Kit 1.0 following the manufacturer ‘s recommended microbial multiplexing protocol. Each set of 4 samples was sequenced using the Pacific Biosciences (PacBio) Sequel platform (v2.1 Chemistry, Sequel SMRT cell 1 M v2, 10-hour movie). The PacBio raw reads were de novo assembled using the PacBio Hierarchical Genome Assembly Process (HGAP) 4.0. The assembled genomes were circularized using Circlator [8]. If the corresponding Illumina short reads were available in NCBI, the closed genomes were further polished with the Illumina short reads using Pilon [9]. In cases where short reads were unavailable, the closed genomes were polished with the PacBio raw reads using the Resequencing module provided by PacBio SMRTLink (from version 5.1 to 8.0) (Pacific Biosciences, Menlo Park, CA).

Among the 217 closed genomes, CFSAN017963 and CFSAN000871 were sequenced on PacbBio RS II, assembled using HGAP 3.0, and published previously [10, 11]. They were re-sequenced on PacBio Sequel which, compared to RS II, provided higher throughput and higher consensus accuracy, assembled using HGAP 4.0, and included in the current dataset for comparison purpose.

**Table 1:** Complete DNA sequences (.Fasta) for the 217 Salmonella genomes

Label	Name of data file/ data set	Data repository and identifier (DOI or accession number)
Data set 1	S. Enteritidis CFSAN000045	NCBI: https://identifiers.org/ncbi/insdc.gca:GCA_018338875.1 [12]
Data set 2	S. Bareilly CFSAN000224	NCBI: https://identifiers.org/ncbi/insdc.gca:GCA_018338855.1 [12]
Data set 3	S. Heidelberg CFSAN000445	NCBI: https://identifiers.org/ncbi/insdc.gca:GCA_018338435.1 [12]
Data set 4	S. 66:z41:- CFSAN000510	NCBI: https://identifiers.org/ncbi/insdc.gca:GCA_018338735.1 [12]
Data set 5	S. Anatum CFSAN000511	NCBI: https://identifiers.org/ncbi/insdc.gca:GCA_018338835.1 [12]
Data set 6	S. Choleraesuis CFSAN000514	NCBI: https://identifiers.org/ncbi/insdc.gca:GCA_018338815.1 [12]
Data set 7	S. Dublin CFSAN000516	NCBI: https://identifiers.org/ncbi/insdc.gca:GCA_018338715.1 [12]
Data set 8	S. Dublin CFSAN000517	NCBI: https://identifiers.org/ncbi/insdc.gca:GCA_018338555.1 [12]
Data set 9	S. Indiana CFSAN000520	NCBI: https://identifiers.org/ncbi/insdc.gca:GCA_007232625.2 [12]
Data set 10	S. Paratyphi B CFSAN000535	NCBI: https://identifiers.org/ncbi/insdc.gca:GCA_010732035.2 [12]
Data set 11	S. Paratyphi B CFSAN000540	NCBI: https://identifiers.org/ncbi/insdc.gca:GCA_003869075.2 [12]
Data set 12	S. Paratyphi B CFSAN000541	NCBI: https://identifiers.org/ncbi/insdc.gca:GCA_006519455.2 [12]
Data set 13	S. Paratyphi B CFSAN000542	NCBI: https://identifiers.org/ncbi/insdc.gca:GCA_006518555.2 [12]
Data set 14	S. Paratyphi B CFSAN000545	NCBI: https://identifiers.org/ncbi/insdc.gca:GCA_018338795.1 [12]
Data set 15	S. Paratyphi B CFSAN000546	NCBI: https://identifiers.org/ncbi/insdc.gca:GCA_018338775.1 [12]
Data set 16	S. Paratyphi B CFSAN000548	NCBI: https://identifiers.org/ncbi/insdc.gca:GCA_018338755.1 [12]
Data set 17	S. Paratyphi B CFSAN000549	NCBI: https://identifiers.org/ncbi/insdc.gca:GCA_018338695.1 [12]
Data set 18	S. 40:z4,z24:- CFSAN000554	NCBI: https://identifiers.org/ncbi/insdc.gca:GCA_018338515.1 [12]
Data set 19	S. Derby CFSAN000565	NCBI: https://identifiers.org/ncbi/insdc.gca:GCA_018338675.1 [12]
Data set 20	S. Newport CFSAN000598	NCBI: https://identifiers.org/ncbi/insdc.gca:GCA_018338635.1 [12]
Data set 21	S. Gallinarum CFSAN000606	NCBI: https://identifiers.org/ncbi/insdc.gca:GCA_018338655.1 [12]
Data set 22	S. Saintpaul CFSAN000614	NCBI: https://identifiers.org/ncbi/insdc.gca:GCA_018338535.1 [12]
Data set 23	S. Typhimurium CFSAN000630	NCBI: https://identifiers.org/ncbi/insdc.gca:GCA_003871895.2 [12]
Data set 24	S. Typhimurium CFSAN000646	NCBI: https://identifiers.org/ncbi/insdc.gca:GCA_007762235.2 [12]
Data set 25	S. Typhimurium CFSAN000648	NCBI: https://identifiers.org/ncbi/insdc.gca:GCA_004222645.2 [12]
Data set 26	S. Wien CFSAN000656	NCBI: https://identifiers.org/ncbi/insdc.gca:GCA_018338415.1 [12]
Data set 27	S. Abaetetuba CFSAN000658	NCBI: https://identifiers.org/ncbi/insdc.gca:GCA_018338375.2 [12]
Data set 28	S. Panama CFSAN000713	NCBI: https://identifiers.org/ncbi/insdc.gca:GCA_018338495.1 [12]
Data set 29	S. Thompson CFSAN000736	NCBI: https://identifiers.org/ncbi/insdc.gca:GCA_018338475.1 [12]
Data set 30	S. Bareilly CFSAN000755	NCBI: https://identifiers.org/ncbi/insdc.gca:GCA_018338355.1 [12]
Data set 31	S. Newport CFSAN000827	NCBI: https://identifiers.org/ncbi/insdc.gca:GCA_003581245.2 [12]
Data set 32	S. Newport CFSAN000871	NCBI: https://identifiers.org/ncbi/insdc.gca:GCA_018338455.2 [12]
Data set 33	S. Javiana CFSAN000905	NCBI: https://identifiers.org/ncbi/insdc.gca:GCA_018338395.1 [12]
Data set 34	S. Typhimurium CFSAN000982	NCBI: https://identifiers.org/ncbi/insdc.gca:GCA_008916265.2 [12]
Data set 35	S. Typhi CFSAN001004	NCBI: https://identifiers.org/ncbi/insdc.gca:GCA_011117275.2 [12]
Data set 36	S. Brandenburg CFSAN001008	NCBI: https://identifiers.org/ncbi/insdc.gca:GCA_007230335.2 [12]
Data set 37	S. II 47:d: z39 CFSAN001010	NCBI: https://identifiers.org/ncbi/insdc.gca:GCA_005899965.2 [12]
Data set 38	S. II 48:d: z6 CFSAN001011	NCBI: https://identifiers.org/ncbi/insdc.gca:GCA_006914355.2 [12]
Data set 39	S. II 50:b: z6 CFSAN001013	NCBI: https://identifiers.org/ncbi/insdc.gca:GCA_006912375.2 [12]
Data set 40	S. II 13,22:z29:e, n,x CFSAN001015	NCBI: https://identifiers.org/ncbi/insdc.gca:GCA_005900065.2 [12]
Data set 41	S. II 4:b:- CFSAN001016	NCBI: https://identifiers.org/ncbi/insdc.gca:GCA_003873385.2 [12]
Data set 42	S. IIIa 41:z4,z23:- CFSAN001018	NCBI: https://identifiers.org/ncbi/insdc.gca:GCA_004222725.2 [12]
Data set 43	S. 40:z35:- CFSAN001045	NCBI: https://identifiers.org/ncbi/insdc.gca:GCA_014338425.3 [12]
Data set 44	S. Senftenberg CFSAN001066	NCBI: https://identifiers.org/ncbi/insdc.gca:GCA_009221265.2 [12]
Data set 45	S. Montevideo CFSAN001068	NCBI: https://identifiers.org/ncbi/insdc.gca:GCA_009221365.2 [12]
Data set 46	S. Javiana CFSAN001070	NCBI: https://identifiers.org/ncbi/insdc.gca:GCA_018338275.1 [12]
Data set 47	S. London CFSAN001081	NCBI: https://identifiers.org/ncbi/insdc.gca:GCA_018338335.1 [12]
Data set 48	S. Havana CFSAN001082	NCBI: https://identifiers.org/ncbi/insdc.gca:GCA_018338295.1 [12]
Data set 49	S. Cubana CFSAN001083	NCBI: https://identifiers.org/ncbi/insdc.gca:GCA_018338315.1 [12]
Data set 50	S. Eastbourne CFSAN001084	NCBI: https://identifiers.org/ncbi/insdc.gca:GCA_018338255.1 [12]
Data set 51	S. Nchanga CFSAN001091	NCBI: https://identifiers.org/ncbi/insdc.gca:GCA_018340155.1 [12]
Data set 52	S. Saintpaul CFSAN001195	NCBI: https://identifiers.org/ncbi/insdc.gca:GCA_000486545.2 [12]
Data set 53	S. Meleagridis CFSAN001281	NCBI: https://identifiers.org/ncbi/insdc.gca:GCA_018339875.1 [12]
Data set 54	S. Give CFSAN001314	NCBI: https://identifiers.org/ncbi/insdc.gca:GCA_000487215.2 [12]
Data set 55	S. Derby CFSAN001315	NCBI: https://identifiers.org/ncbi/insdc.gca:GCA_018340175.1 [12]
Data set 56	S. Soerenga CFSAN001321	NCBI: https://identifiers.org/ncbi/insdc.gca:GCA_018340135.1 [12]
Data set 57	S. Cerro CFSAN001330	NCBI: https://identifiers.org/ncbi/insdc.gca:GCA_000487275.2 [12]
Data set 58	S. Miami CFSAN001416	NCBI: https://identifiers.org/ncbi/insdc.gca:GCA_018339975.1 [12]
Data set 59	S. Javiana CFSAN001999	NCBI: https://identifiers.org/ncbi/insdc.gca:GCA_014447715.2 [12]
Data set 60	S. Typhimurium CFSAN002003	NCBI: https://identifiers.org/ncbi/insdc.gca:GCA_019457675.1 [12]
Data set 61	S. Anatum CFSAN003983	NCBI: https://identifiers.org/ncbi/insdc.gca:GCA_018339915.1 [12]
Data set 62	S. Anatum CFSAN003985	NCBI: https://identifiers.org/ncbi/insdc.gca:GCA_010542265.2 [12]
Data set 63	S. Saintpaul CFSAN004079	NCBI: https://identifiers.org/ncbi/insdc.gca:GCA_018340095.1 [12]
Data set 64	S. Saintpaul CFSAN004085	NCBI: https://identifiers.org/ncbi/insdc.gca:GCA_018340075.1 [12]
Data set 65	S. Saintpaul CFSAN004090	NCBI: https://identifiers.org/ncbi/insdc.gca:GCA_018339995.1 [12]
Data set 66	S. Saintpaul CFSAN004112	NCBI: https://identifiers.org/ncbi/insdc.gca:GCA_018339955.1 [12]
Data set 67	S. Saintpaul CFSAN004114	NCBI: https://identifiers.org/ncbi/insdc.gca:GCA_018339895.1 [12]
Data set 68	S. Give CFSAN004343	NCBI: https://identifiers.org/ncbi/insdc.gca:GCA_018339855.2 [12]
Data set 69	S. Muenster CFSAN004344	NCBI: https://identifiers.org/ncbi/insdc.gca:GCA_018339495.2 [12]
Data set 70	S. Minnesota CFSAN006156	NCBI: https://identifiers.org/ncbi/insdc.gca:GCA_018339775.1 [12]
Data set 71	S. Saintpaul CFSAN006166	NCBI: https://identifiers.org/ncbi/insdc.gca:GCA_018339795.1 [12]
Data set 72	S. Rubislaw CFSAN006171	NCBI: https://identifiers.org/ncbi/insdc.gca:GCA_018339755.1 [12]
Data set 73	S. Anatum CFSAN006191	NCBI: https://identifiers.org/ncbi/insdc.gca:GCA_018339715.1 [12]
Data set 74	S. Saintpaul CFSAN006195	NCBI: https://identifiers.org/ncbi/insdc.gca:GCA_018339515.1 [12]
Data set 75	S. Braenderup CFSAN006197	NCBI: https://identifiers.org/ncbi/insdc.gca:GCA_018339535.1 [12]
Data set 76	S. Senftenberg CFSAN006211	NCBI: https://identifiers.org/ncbi/insdc.gca:GCA_018339695.1 [12]
Data set 77	S. Braenderup CFSAN006217	NCBI: https://identifiers.org/ncbi/insdc.gca:GCA_018339675.1 [12]
Data set 78	S. Javiana CFSAN006238	NCBI: https://identifiers.org/ncbi/insdc.gca:GCA_018339655.1 [12]
Data set 79	S. Saintpaul CFSAN006241	NCBI: https://identifiers.org/ncbi/insdc.gca:GCA_018339595.1 [12]
Data set 80	S. Saintpaul CFSAN006246	NCBI: https://identifiers.org/ncbi/insdc.gca:GCA_018339475.1 [12]
Data set 81	S. Agona CFSAN006264	NCBI: https://identifiers.org/ncbi/insdc.gca:GCA_018339615.1 [12]
Data set 82	S. Javiana CFSAN006269	NCBI: https://identifiers.org/ncbi/insdc.gca:GCA_018339635.1 [12]
Data set 83	S. Typhimurium CFSAN008081	NCBI: https://identifiers.org/ncbi/insdc.gca:GCA_001756025.2 [12]
Data set 84	S. Enteritidis CFSAN008104	NCBI: https://identifiers.org/ncbi/insdc.gca:GCA_001713515.2 [12]
Data set 85	S. Buzu CFSAN008300	NCBI: https://identifiers.org/ncbi/insdc.gca:GCA_018339555.1 [12]
Data set 86	S. Paratyphi B CFSAN008708	NCBI: https://identifiers.org/ncbi/insdc.gca:GCA_003878535.2 [12]
Data set 87	S. Hartford CFSAN008725	NCBI: https://identifiers.org/ncbi/insdc.gca:GCA_014432465.2 [12]
Data set 88	S. Hartford CFSAN008740	NCBI: https://identifiers.org/ncbi/insdc.gca:GCA_018339455.1 [12]
Data set 89	S. Sharon CFSAN008783	NCBI: https://identifiers.org/ncbi/insdc.gca:GCA_006949785.2 [12]
Data set 90	S. Inverness CFSAN008785	NCBI: https://identifiers.org/ncbi/insdc.gca:GCA_005411755.2 [12]
Data set 91	S. Inverness CFSAN008789	NCBI: https://identifiers.org/ncbi/insdc.gca:GCA_006949085.2 [12]
Data set 92	S. Muenchen CFSAN008798	NCBI: https://identifiers.org/ncbi/insdc.gca:GCA_014431865.2 [12]
Data set 93	S. Inverness CFSAN008810	NCBI: https://identifiers.org/ncbi/insdc.gca:GCA_006948385.2 [12]
Data set 94	S. Inverness CFSAN008812	NCBI: https://identifiers.org/ncbi/insdc.gca:GCA_005534535.2 [12]
Data set 95	S. Inverness CFSAN008813	NCBI: https://identifiers.org/ncbi/insdc.gca:GCA_005991765.2 [12]
Data set 96	S. Schwarzengrund CFSAN008848	NCBI: https://identifiers.org/ncbi/insdc.gca:GCA_018340505.1 [12]
Data set 97	S. Kentucky CFSAN011778	NCBI: https://identifiers.org/ncbi/insdc.gca:GCA_018340485.1 [12]
Data set 98	S. Montevideo CFSAN012296	NCBI: https://identifiers.org/ncbi/insdc.gca:GCA_018340465.1 [12]
Data set 99	S. Infantis CFSAN012496	NCBI: https://identifiers.org/ncbi/insdc.gca:GCA_008025235.2 [12]
Data set 100	S. Oranienburg CFSAN012497	NCBI: https://identifiers.org/ncbi/insdc.gca:GCA_010651985.2 [12]
Data set 101	S. Sandiego CFSAN012498	NCBI: https://identifiers.org/ncbi/insdc.gca:GCA_011436695.2 [12]
Data set 102	S. Sandiego CFSAN012509	NCBI: https://identifiers.org/ncbi/insdc.gca:GCA_007626775.2 [12]
Data set 103	S. Give CFSAN012622	NCBI: https://identifiers.org/ncbi/insdc.gca:GCA_000732045.2 [12]
Data set 104	S. Derby CFSAN013765	NCBI: https://identifiers.org/ncbi/insdc.gca:GCA_006651685.2 [12]
Data set 105	S. Meleagridis CFSAN017807	NCBI: https://identifiers.org/ncbi/insdc.gca:GCA_007635275.2 [12]
Data set 106	S. Minnesota CFSAN017963	NCBI: https://identifiers.org/ncbi/insdc.gca:GCA_002196535.1 [12]
Data set 107	S. Typhimurium CFSAN018746	NCBI: https://identifiers.org/ncbi/insdc.gca:GCA_003030285.2 [12]
Data set 108	S. Newport CFSAN022621	NCBI: https://identifiers.org/ncbi/insdc.gca:GCA_008606975.2 [12]
Data set 109	S. Newport CFSAN022622	NCBI: https://identifiers.org/ncbi/insdc.gca:GCA_008606925.2 [12]
Data set 110	S. Choleraesuis CFSAN022623	NCBI: https://identifiers.org/ncbi/insdc.gca:GCA_010713575.2 [12]
Data set 111	S. Gallinarum CFSAN022627	NCBI: https://identifiers.org/ncbi/insdc.gca:GCA_007626135.2 [12]
Data set 112	S. Choleraesuis CFSAN022628	NCBI: https://identifiers.org/ncbi/insdc.gca:GCA_010396085.2 [12]
Data set 113	S. Choleraesuis CFSAN022631	NCBI: https://identifiers.org/ncbi/insdc.gca:GCA_010794875.2 [12]
Data set 114	S. Newport CFSAN022633	NCBI: https://identifiers.org/ncbi/insdc.gca:GCA_008607025.2 [12]
Data set 115	S. Dublin CFSAN022635	NCBI: https://identifiers.org/ncbi/insdc.gca:GCA_008607005.2 [12]
Data set 116	S. Enteritidis CFSAN022640	NCBI: https://identifiers.org/ncbi/insdc.gca:GCA_006651425.2 [12]
Data set 117	S. Gallinarum CFSAN022642	NCBI: https://identifiers.org/ncbi/insdc.gca:GCA_007500235.2 [12]
Data set 118	S. Gallinarum CFSAN024144	NCBI: https://identifiers.org/ncbi/insdc.gca:GCA_005676255.2 [12]
Data set 119	S. Gaminara CFSAN024152	NCBI: https://identifiers.org/ncbi/insdc.gca:GCA_005936085.2 [12]
Data set 120	S. Bovismorbificans CFSAN024184	NCBI: https://identifiers.org/ncbi/insdc.gca:GCA_006846945.2 [12]
Data set 121	S. Bareilly CFSAN024219	NCBI: https://identifiers.org/ncbi/insdc.gca:GCA_005935865.2 [12]
Data set 122	S. Give CFSAN024230	NCBI: https://identifiers.org/ncbi/insdc.gca:GCA_006849305.2 [12]
Data set 123	S. Newport CFSAN024414	NCBI: https://identifiers.org/ncbi/insdc.gca:GCA_018340235.1 [12]
Data set 124	S. Newport CFSAN024415	NCBI: https://identifiers.org/ncbi/insdc.gca:GCA_018340215.1 [12]
Data set 125	S. Newport CFSAN024417	NCBI: https://identifiers.org/ncbi/insdc.gca:GCA_018340425.1 [12]
Data set 126	S. Tennessee CFSAN024439	NCBI: https://identifiers.org/ncbi/insdc.gca:GCA_037201925.1 [12]
Data set 127	S. Gaminara CFSAN024441	NCBI: https://identifiers.org/ncbi/insdc.gca:GCA_008730735.2 [12]
Data set 128	S. Newport CFSAN024495	NCBI: https://identifiers.org/ncbi/insdc.gca:GCA_037347515.1 [12]
Data set 129	S. Newport CFSAN024515	NCBI: https://identifiers.org/ncbi/insdc.gca:GCA_018340355.1 [12]
Data set 130	S. Muenchen CFSAN024517	NCBI: https://identifiers.org/ncbi/insdc.gca:GCA_018340335.1 [12]
Data set 131	S. Muenchen CFSAN024522	NCBI: https://identifiers.org/ncbi/insdc.gca:GCA_018340275.1 [12]
Data set 132	S. Newport CFSAN024541	NCBI: https://identifiers.org/ncbi/insdc.gca:GCA_018340315.1 [12]
Data set 133	S. Weltevreden CFSAN024549	NCBI: https://identifiers.org/ncbi/insdc.gca:GCA_018340295.1 [12]
Data set 134	S. Pomona CFSAN024552	NCBI: https://identifiers.org/ncbi/insdc.gca:GCA_037345635.1 [12]
Data set 135	S. Newport CFSAN024555	NCBI: https://identifiers.org/ncbi/insdc.gca:GCA_018340255.1 [12]
Data set 136	S. Johannesburg CFSAN024562	NCBI: https://identifiers.org/ncbi/insdc.gca:GCA_018340525.1 [12]
Data set 137	S. Saintpaul CFSAN024564	NCBI: https://identifiers.org/ncbi/insdc.gca:GCA_018340195.1 [12]
Data set 138	S. Irumu CFSAN024579	NCBI: https://identifiers.org/ncbi/insdc.gca:GCA_018339415.1 [12]
Data set 139	S. Rubislaw CFSAN024580	NCBI: https://identifiers.org/ncbi/insdc.gca:GCA_018339375.1 [12]
Data set 140	S. Minnesota CFSAN024581	NCBI: https://identifiers.org/ncbi/insdc.gca:GCA_018338975.1 [12]
Data set 141	S. Rubislaw CFSAN024587	NCBI: https://identifiers.org/ncbi/insdc.gca:GCA_018339055.1 [12]
Data set 142	S. Sandiego CFSAN024590	NCBI: https://identifiers.org/ncbi/insdc.gca:GCA_018339355.1 [12]
Data set 143	S. Newport CFSAN024599	NCBI: https://identifiers.org/ncbi/insdc.gca:GCA_018339175.1 [12]
Data set 144	S. Newport CFSAN024608	NCBI: https://identifiers.org/ncbi/insdc.gca:GCA_018339255.1 [12]
Data set 145	S. Waycross CFSAN024659	NCBI: https://identifiers.org/ncbi/insdc.gca:GCA_006858285.2 [12]
Data set 146	S. Livingstone CFSAN024717	NCBI: https://identifiers.org/ncbi/insdc.gca:GCA_000807055.1 [12]
Data set 147	S. Senftenberg CFSAN024719	NCBI: https://www.ncbi.nlm.nih.gov/nuccore/CP074261.1 [12]
Data set 148	S. Senftenberg CFSAN024722	NCBI: https://www.ncbi.nlm.nih.gov/nuccore/CP074260.1 [12]
Data set 149	S. Anatum CFSAN024763	NCBI: https://www.ncbi.nlm.nih.gov/nuccore/CP074259.1 [12]
Data set 150	S. Brandenburg CFSAN024765	NCBI: https://www.ncbi.nlm.nih.gov/nuccore/CP074258.1 [12]
Data set 151	S. Infantis CFSAN024778	NCBI: https://www.ncbi.nlm.nih.gov/nuccore/CP074261.1 [12]
Data set 152	S. Infantis CFSAN024781	NCBI: https://www.ncbi.nlm.nih.gov/nuccore/CP074256.1 [12]
Data set 153	S. Enteritidis CFSAN026631	NCBI: https://identifiers.org/ncbi/insdc.gca:GCA_018339215.1 [12]
Data set 154	S. Enteritidis CFSAN026633	NCBI: https://identifiers.org/ncbi/insdc.gca:GCA_018339155.1 [12]
Data set 155	S. Rubislaw CFSAN027379	NCBI: https://identifiers.org/ncbi/insdc.gca:GCA_018339115.1 [12]
Data set 156	S. Braenderup CFSAN027384	NCBI: https://identifiers.org/ncbi/insdc.gca:GCA_018339075.1 [12]
Data set 157	S. Kentucky CFSAN027385	NCBI: https://identifiers.org/ncbi/insdc.gca:GCA_018339035.1 [12]
Data set 158	S. Kentucky CFSAN027395	NCBI: https://identifiers.org/ncbi/insdc.gca:GCA_018338955.1 [12]
Data set 159	S. Brandenburg CFSAN027396	NCBI: https://identifiers.org/ncbi/insdc.gca:GCA_018338995.1 [12]
Data set 160	S. Montevideo CFSAN028508	NCBI: https://identifiers.org/ncbi/insdc.gca:GCA_018339015.1 [12]
Data set 161	S. Kentucky CFSAN028527	NCBI: https://identifiers.org/ncbi/insdc.gca:GCA_018338935.1 [12]
Data set 162	S. Weltevreden CFSAN028546	NCBI: https://identifiers.org/ncbi/insdc.gca:GCA_010699505.2 [12]
Data set 163	S. Oranienburg CFSAN028548	NCBI: https://identifiers.org/ncbi/insdc.gca:GCA_003872475.2 [12]
Data set 164	S. Newport CFSAN028549	NCBI: https://identifiers.org/ncbi/insdc.gca:GCA_008029255.2 [12]
Data set 165	S. Shamba CFSAN029516	NCBI: https://identifiers.org/ncbi/insdc.gca:GCA_007473075.2 [12]
Data set 166	S. Potsdam CFSAN029622	NCBI: https://identifiers.org/ncbi/insdc.gca:GCA_018338915.1 [12]
Data set 167	S. Worthington CFSAN029662	NCBI: https://identifiers.org/ncbi/insdc.gca:GCA_005899025.2 [12]
Data set 168	S. Gaminara CFSAN029843	NCBI: https://identifiers.org/ncbi/insdc.gca:GCA_005606435.2 [12]
Data set 169	S. Saintpaul CFSAN029855	NCBI: https://identifiers.org/ncbi/insdc.gca:GCA_006016765.2 [12]
Data set 170	S. Rubislaw CFSAN029856	NCBI: https://identifiers.org/ncbi/insdc.gca:GCA_006868345.2 [12]
Data set 171	S. Rubislaw CFSAN029865	NCBI: https://identifiers.org/ncbi/insdc.gca:GCA_006868405.2 [12]
Data set 172	S. Reading CFSAN029868	NCBI: https://identifiers.org/ncbi/insdc.gca:GCA_005946705.2 [12]
Data set 173	S. Muenchen CFSAN029871	NCBI: https://identifiers.org/ncbi/insdc.gca:GCA_004223785.2 [12]
Data set 174	S. Rubislaw CFSAN029872	NCBI: https://identifiers.org/ncbi/insdc.gca:GCA_005532935.2 [12]
Data set 175	S. Mississippi CFSAN029878	NCBI: https://identifiers.org/ncbi/insdc.gca:GCA_006868905.2 [12]
Data set 176	S. Inverness CFSAN029879	NCBI: https://identifiers.org/ncbi/insdc.gca:GCA_005532895.2 [12]
Data set 177	S. Gaminara CFSAN029882	NCBI: https://identifiers.org/ncbi/insdc.gca:GCA_006870005.2 [12]
Data set 178	S. Inverness CFSAN029891	NCBI: https://identifiers.org/ncbi/insdc.gca:GCA_006869965.2 [12]
Data set 179	S. Muenchen CFSAN029894	NCBI: https://identifiers.org/ncbi/insdc.gca:GCA_005606535.2 [12]
Data set 180	S. Inverness CFSAN029895	NCBI: https://identifiers.org/ncbi/insdc.gca:GCA_003872625.2 [12]
Data set 181	S. Newport CFSAN029927	NCBI: https://identifiers.org/ncbi/insdc.gca:GCA_006873825.2 [12]
Data set 182	S. Rubislaw CFSAN029939	NCBI: https://identifiers.org/ncbi/insdc.gca:GCA_003872215.2 [12]
Data set 183	S. Bareilly CFSAN029943	NCBI: https://identifiers.org/ncbi/insdc.gca:GCA_006090915.2 [12]
Data set 184	S. Mississippi CFSAN029945	NCBI: https://identifiers.org/ncbi/insdc.gca:GCA_003872395.2 [12]
Data set 185	S. Typhimurium CFSAN029958	NCBI: https://identifiers.org/ncbi/insdc.gca:GCA_006019175.2 [12]
Data set 186	S. IIIb 61:l, v:z35 CFSAN030538	NCBI: https://identifiers.org/ncbi/insdc.gca:GCA_003872565.2 [12]
Data set 187	S. Oranienburg CFSAN030601	NCBI: https://identifiers.org/ncbi/insdc.gca:GCA_005532075.2 [12]
Data set 188	S. Typhimurium CFSAN033950	NCBI: https://identifiers.org/ncbi/insdc.gca:GCA_001652385.2 [12]
Data set 189	S. IIIb 60:r: e,n, x,z15 CFSAN044865	NCBI: https://identifiers.org/ncbi/insdc.gca:GCA_001831555.2 [12]
Data set 190	S. I 38:k:- CFSAN044875	NCBI: https://identifiers.org/ncbi/insdc.gca:GCA_001831715.2 [12]
Data set 191	S. Meleagridis CFSAN044885	NCBI: https://identifiers.org/ncbi/insdc.gca:GCA_001831915.2 [12]
Data set 192	S. Rubislaw CFSAN044888	NCBI: https://identifiers.org/ncbi/insdc.gca:GCA_001831985.2 [12]
Data set 193	S. Saintpaul CFSAN044909	NCBI: https://identifiers.org/ncbi/insdc.gca:GCA_001833225.2 [12]
Data set 194	S. Inverness CFSAN044911	NCBI: https://identifiers.org/ncbi/insdc.gca:GCA_001834735.2 [12]
Data set 195	S. Rubislaw CFSAN044921	NCBI: https://identifiers.org/ncbi/insdc.gca:GCA_001833255.2 [12]
Data set 196	S. 60:r: e,n, x,z15 CFSAN044923	NCBI: https://identifiers.org/ncbi/insdc.gca:GCA_001832255.2 [12]
Data set 197	S. Saintpaul CFSAN044925	NCBI: https://identifiers.org/ncbi/insdc.gca:GCA_001833305.2 [12]
Data set 198	S. Rubislaw CFSAN044945	NCBI: https://identifiers.org/ncbi/insdc.gca:GCA_001834965.2 [12]
Data set 199	S. Enteritidis CFSAN051827	NCBI: https://identifiers.org/ncbi/insdc.gca:GCA_001973785.2 [12]
Data set 200	S. Enteritidis CFSAN051882	NCBI: https://identifiers.org/ncbi/insdc.gca:GCA_001972625.2 [12]
Data set 201	S. Enteritidis CFSAN051890	NCBI: https://identifiers.org/ncbi/insdc.gca:GCA_001972545.2 [12]
Data set 202	S. Senftenberg CFSAN056662	NCBI: https://identifiers.org/ncbi/insdc.gca:GCA_006344865.2 [12]
Data set 203	S. Eastbourne CFSAN059883	NCBI: https://identifiers.org/ncbi/insdc.gca:GCA_002266205.2 [12]
Data set 204	S. Dublin CFSAN059898	NCBI: https://identifiers.org/ncbi/insdc.gca:GCA_002266085.2 [12]
Data set 205	S. Infantis CFSAN059939	NCBI: https://identifiers.org/ncbi/insdc.gca:GCA_018340565.1 [12]
Data set 206	S. Infantis CFSAN059940	NCBI: https://identifiers.org/ncbi/insdc.gca:GCA_018340545.1 [12]
Data set 207	S. Give CFSAN060804	NCBI: https://identifiers.org/ncbi/insdc.gca:GCA_002507915.2 [12]
Data set 208	S. Uganda CFSAN060807	NCBI: https://identifiers.org/ncbi/insdc.gca:GCA_002507875.2 [12]
Data set 209	S. I 1,3,19:l, v:1,2 CFSAN060808	NCBI: https://identifiers.org/ncbi/insdc.gca:GCA_002507745.2 [12]
Data set 210	S. Manchester CFSAN060809	NCBI: https://identifiers.org/ncbi/insdc.gca:GCA_002507865.2 [12]
Data set 211	S. Anatum CFSAN064276	NCBI: https://identifiers.org/ncbi/insdc.gca:GCA_002507715.2 [12]
Data set 212	S. Javiana CFSAN073546	NCBI: https://identifiers.org/ncbi/insdc.gca:GCA_006199305.2 [12]
Data set 213	S. Oranienburg CFSAN073549	NCBI: https://identifiers.org/ncbi/insdc.gca:GCA_007484345.2 [12]
Data set 214	S. Saintpaul CFSAN073553	NCBI: https://identifiers.org/ncbi/insdc.gca:GCA_006315305.2 [12]
Data set 215	S. Javiana CFSAN073554	NCBI: https://identifiers.org/ncbi/insdc.gca:GCA_006199285.2 [12]
Data set 216	S. Muenchen CFSAN073555	NCBI: https://identifiers.org/ncbi/insdc.gca:GCA_007634455.2 [12]
Data set 217	S. Mbandaka CFSAN082805	NCBI: https://identifiers.org/ncbi/insdc.gca:GCA_006520715.2 [12]

## Data Availability

Data is provided within the manuscript (Table 1), which have been deposited in NCBI with the primary accession number PRJNA186035.

## Abbreviations

FDA-CFSAN: U.S. Food and Drug Administration’s Center for Food Safety and Applied Nutrition
NCBI: National Center for Biotechnology Information
SNP: Single Nucleotide Polymorphism
SRA: Short-read Archive
WGS: Whole Genome Sequencing
